# Nitric Oxide Metabolites and Lung Cancer Incidence: A Matched Case-Control Study Nested in the ESTHER Cohort

**DOI:** 10.1155/2019/6470950

**Published:** 2019-09-02

**Authors:** Xīn Gào, Yang Xuan, Axel Benner, Ankita Anusruti, Hermann Brenner, Ben Schöttker

**Affiliations:** ^1^Division of Clinical Epidemiology and Aging Research, German Cancer Research Center, Heidelberg, Germany; ^2^Network Aging Research, Heidelberg University, Germany; ^3^Division of Biostatistics, German Cancer Research Center, Heidelberg, Germany; ^4^Division of Preventive Oncology, German Cancer Research Center and National Center for Tumor Diseases (NCT), Heidelberg, Germany; ^5^German Cancer Consortium (DKTK), German Cancer Research Center, Heidelberg, Germany

## Abstract

Studies suggest that nitric oxide (NO) may have a possible role in lung carcinogenesis. This study is aimed to evaluate the association of the NO metabolites, namely, nitrite and nitrate, with lung cancer incidence. We conducted a matched case-control study (*n* = 245 incident lung cancer cases and *n* = 735 controls) based on the German ESTHER cohort (*n* = 9,940). Controls were matched to cases on age, sex, smoking status (never/former/current smoking), and pack-years of smoking. The sum of nitrite and nitrate was measured in urine samples using a colorimetric assay and was standardized for renal function by urinary creatinine. Conditional logistic regression models, adjusted for lifestyle factors, asthma prevalence, and family history of lung cancer, were used to estimate odds ratios (ORs) and 95% confidence intervals (95% CI). Among incident lung cancer cases, high nitrite/nitrate levels were statistically significantly associated with current smoking, a low BMI, and the oxidative stress biomarker 8-isoprostane levels. Nitrite/nitrate levels in the top quintile were statistically significantly associated with lung cancer incidence: the OR (95% CI) was 1.37 (1.04-1.82) for comparison with the bottom quintile. This association was unaltered after additional adjustment for 8-isoprostane levels and C-reactive protein (CRP). In conclusion, this large cohort study suggested that subjects with high urinary nitrite/nitrate concentrations had an increased risk of lung cancer and this association was independent of smoking, CRP, 8-isoprostane levels, and other established lung cancer risk factors. Further studies are needed to validate these findings and to confirm the hypothesis that pathologically high levels of NO are involved in lung cancer development.

## 1. Introduction

Lung cancer is one of the most common causes of cancer death worldwide with a poor prognosis [1, 2]. Oxidative stress is suggested to mediate chronic inflammation-induced lung cancer development [3]. Inflammatory cells are recruited to the site of inflammation leading to respiratory burst, during which the inflammatory cells produce more reactive oxygen species (ROS). The sustained oxidative environment results in the transformation of normal cells to cancer cells [4, 5]. In addition, smokers have a much higher risk of developing lung cancer than nonsmokers [6]. It has been shown that cigarette smoking leads to oxidative stress and inflammation in an acute cigarette smoking model [7]. Therefore, oxidative stress and inflammation might partially mediate the effect of smoking on lung cancer development.

Endogenous nitric oxide (NO) is a multifunctional inflammatory molecule and promotes inflammation under physiological condition [8]. It is synthesized by 3 isoforms of NO synthase (NOS). The neuronal NOS and endothelial NOS constitutively catalyze the formation of NO, while the inducible NOS (iNOS) is being induced by inflammatory cytokines and produces larger, toxic amounts of NO [9]. NO is also a free radical. It has one unpaired electron, which makes it susceptible for reactions with other radical species [10]. For instance, the reaction of NO with the superoxide anion (O_2_^−^) leads to peroxynitrite (ONOO^−^) formation [11]. Thus, excessive formation of NO results in elevated generation of reactive oxygen/nitrogen species, which may induce oxidative stress.

NO plays a pivotal role in cancer development. On the one hand, excessive NO is toxic and can prevent tumor growth by increasing the apoptosis rate of cells. While on the other hand, NO is a mediator of signaling pathways, which promote cancer progression and metastasis [9]. In summary, NO plays an important role in the complex interrelationships of ROS, inflammation, and cancer development and growth [12].

However, it is difficult to detect NO in tissues and biological fluids directly due to its highly reactive nature and low concentration. The end-products of NO metabolism, nitrite and nitrate, are much more stable and can be used to reflect the systemic NO production [11]. To our knowledge, no previous study has investigated the association between nitrite/nitrate levels and lung cancer development in a prospective, population-based study. We addressed this research question by performing a matched case-control study nested in a large, prospective cohort study from Germany.

## 2. Materials and Methods

### 2.1. Study Population

This investigation was based on the ESTHER cohort study (Epidemiologische Studie zu Chancen der Verhütung, Früherkennung und optimierten Therapie chronischer Erkrankungen in der älteren Bevölkerung [German]), which is a population-based, longitudinal study, with repeated investigation of inhabitants in the German federal state Saarland. The details of the ESTHER cohort study have been reported elsewhere [13]. In brief, 9940 individuals, aged 50-75 years, were invited to participate during a routine health checkup between 2000 and 2002. So far, those participants were recontacted 5 times: 2, 5, 8, 11, and 14 years after baseline. Information on sociodemographic characteristics, lifestyle, smoking habits, and diet was investigated by using a comprehensive questionnaire at baseline as well as at follow-ups. The ESTHER study was approved by the ethics committees of the University of Heidelberg and the state medical board of Saarland, Germany. Written informed consent was issued by all participants.

### 2.2. Cancer Follow-Up

Information on lung cancer was provided by the Saarland Cancer Registry up to the end of the year 2014. Linkage of ESTHER participants with data of the Saarland Cancer Registry was possible for 99.7% of the cohort's participants. Lung cancer cases were ascertained according to the 10^th^ revision of the International Statistical Classification of Diseases (ICD-10) code C34.

### 2.3. Selection of Case and Control Subjects

From 252 incident lung cancer cases, during a mean follow-up time of 13.4 years, 245 could be included in the present analysis because they donated a blood sample and a urine sample at baseline of the ESTHER cohort. Each of the lung cancer cases was matched with three controls from the same cohort on sex, age (±5 years), smoking status (never/former/current smoker), and pack-years of smoking (±10 years).

### 2.4. Laboratory Analyses

At baseline, a blood sample and a spontaneous spot urine sample were collected by general practitioners (GPs) during the health checkup and then shipped to the study center and maintained at -80°C until further processing. Urinary concentrations of nitrite/nitrate were determined using the nitrite/nitrate Colorimetric Assay Kit of Cayman Chemical (Ann Arbor, Michigan, USA). This method detects the sum of nitrate and nitrite. Urine samples were used directly after dilution to a proper concentration (1 : 5, 1 : 25, or 1 : 50 depending on the levels of nitrite/nitrate in the urine sample). For renal function adjustment of the spot urine samples, urinary creatinine was determined by the kinetic Jaffe method. In addition, the acute-phase inflammatory protein C-reactive protein (CRP) was measured in serum samples by immunoturbidimetry with the wrCRP antibody (Bayer, Leverkusen, Germany) on the ADVIA 2400. Furthermore, the levels of an established oxidative stress marker, urinary 8-isoprostane, were determined by the 8iso1 ELISA kit from Detroit R&D (Detroit, Michigan, USA).

### 2.5. Covariates

Information on sociodemographic characteristics, including age, sex, smoking status, pack-years of smoking, education, physical activity, vegetable consumption, meat consumption, family history of lung cancer, and individual history of asthma, was collected by a standardized self-administered questionnaire. To calculate body mass index (BMI), height and weight were measured by the GPs during the health checkup and documented on a standardized form.

### 2.6. Statistical Analyses

Baseline characteristics of cases and controls were expressed as medians (interquartile ranges) or proportions. Differences between the two groups were determined by the Wilcoxon tests for continuous variables and by the chi-square tests for categorical variables. To assess the determinants of nitrite/nitrate concentrations, distributions of nitrite/nitrate concentrations across categories of baseline characteristics were compared using the Wilcoxon-Mann-Whitney tests.

To evaluate the association of nitrite/nitrate levels and lung cancer incidence, conditional logistic regression was used to compute odds ratios (ORs) and 95% confidence intervals (CIs). The main model was adjusted for body mass index (BMI), education, family history of lung cancer, asthma prevalence, physical activity, and vegetable and meat consumption. In sensitivity models, the inflammatory marker CRP and the oxidative stress marker 8-isoprostane were additionally added to the main model. In order to address possible reverse causality, a sensitivity analysis was conducted, in which cancer cases that occurred in the first 5 years of follow-up were excluded. A dose-response analysis was conducted using restricted cubic spline (RCS) functions with five knots at the 10^th^, 30^th^, 50^th^, 70^th^, and 90^th^ percentiles of the nitrite/nitrate distribution.

Multiple imputation was applied to impute covariates with missing values [14]. All covariates had less than 3.7% missing values in study population. Briefly, five data sets were imputed with the SAS procedure PROC MI. The conditional logistic regression models were performed in the five imputed data sets, and the results were combined by the SAS procedure PROC MIANALYZE. Restricted cubic splines were produced from one of the five imputed data sets.

All statistical tests were two-sided using a significant level of 0.05. All analyses were performed with the Statistical Analysis System (SAS) version 9.4 (SAS Institute Inc., Cary, NC).

## 3. Results

Table 1 presents the sociodemographic and lifestyle characteristics of the 245 lung cancer patients and the 735 controls at baseline. Age, sex, smoking status, and pack-years of smoking were almost identically distributed among cases and controls due to the matching. In addition, there was no statistically significant difference between cases and controls in school education, physical activity, vegetable and meat consumption frequency, asthma prevalence, family history of lung cancer, and CRP and 8-isoprostane levels. This is likely also a result of the matching by age, sex, and smoking. The only statistically significantly difference between cases and controls was observed for BMI. Study participants who developed lung cancer during follow-up were more frequently lean (BMI < 25 kg/m^2^) and less frequently obese (BMI > 30 kg/m^2^) compared to matched control subjects. Nitrite/nitrate levels were higher in study participants who were diagnosed with lung cancer during follow-up (median (IQR) = 122 (80‐206)) than among controls (median (IQR) = 114 (75‐170)), but the median difference was not statistically significant.

Table 2 shows median (IQR) nitrite/nitrate levels across the categories of baseline characteristics. High nitrite/nitrate levels were statistically significantly associated with current smoking and high 8-isoprostane concentrations among cases and controls. Among cases, they were additionally statistically significantly associated with a low BMI. Among controls, nitrite/nitrate levels were statistically significantly higher among subjects aged < 65 years compared to older subjects.

Table 3 presents the association between nitrite/nitrate levels and lung cancer incidence. The top quintile of nitrite/nitrate levels (≥192.8 *μ*mol/mmol creatinine) was statistically significantly associated with lung cancer incidence, when compared to the bottom quintile (<66.9 *μ*mol/mmol creatinine): the OR (95% CI) was 1.37 (1.04-1.82) in the main model. Additional adjustments for CRP and 8-isoprostane levels did not change this finding. Furthermore, the strength of the observed association remained similar after excluding lung cancer cases within the first 5 years of follow-up (Table 4).

**Figure 1:**
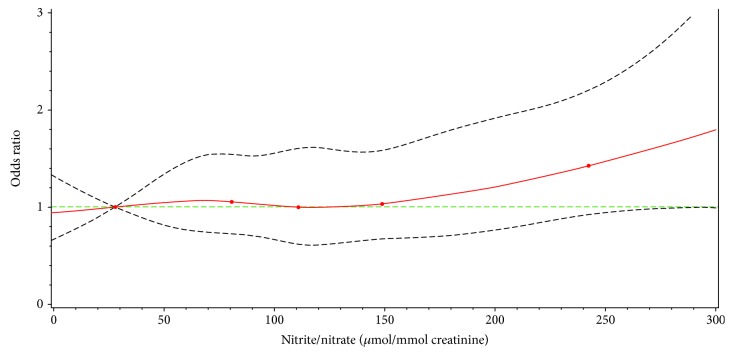
Dose-response relationship of a nitrite/nitrate concentration and lung cancer incidence, the ESTHER study (2000-2014). Notes: results of a logistic regression model adjusted for BMI, education, family history of lung cancer, asthma, physical activity, and vegetable and meat consumption frequency. In addition, potential confounding by the following factors was controlled by matching age, sex, smoking status, and pack-years of smoking. Knots: 10^th^, 30^th^, 50^th^, 70^th^, and 90^th^ percentile. Solid red line: estimation for the odds ratio. Dashed grey lines: 95% confidence interval bands. Dashed green line: odds ratio = 1 as reference.

The dose-response relationship between nitrite/nitrate concentrations and lung cancer incidence is presented in Figure 1. It shows that the association is not linear and that a statistically significant association is only present at high nitrite/nitrate concentrations (approx. >200 *μ*mol/mmol creatinine). This is in agreement with the findings from the logistic models, in which only an increased lung cancer risk was found in the highest nitrite/nitrate quintile (≥192.8 *μ*mol/mmol creatinine).

## 4. Discussion

In this prospective matched case-control study from Germany, we investigated the determinants of urinary nitrite/nitrate levels and the association between nitrite/nitrate levels and lung cancer incidence. We observed that high nitrite/nitrate levels were associated with lower age in controls and a lower BMI among cases. Current smoking and high 8-isoprostane levels were associated with high nitrite/nitrate levels in both groups. Furthermore, the association of nitrite/nitrate levels was not linear, and only participants with nitrite/nitrate levels greater than approximately 200 *μ*mol/mmol creatinine had an increased lung cancer risk.

Previous studies have demonstrated that NO is a mediator and regulator in inflammatory responses. Whereas low NO is anti-inflammatory, excessively elevated NO promotes inflammation and oxidative stress under pathological conditions [15, 16]. This could explain why we only observed an association of nitrite/nitrate levels and lung cancer incidence at high nitrite/nitrate concentrations. Interestingly, this association was independent from CRP and 8-isoprostane levels, which suggests that the pathway from NO to lung cancer is independent from the inflammatory and oxidative stress processes that are reflected by these biomarkers.

**Figure 2:**
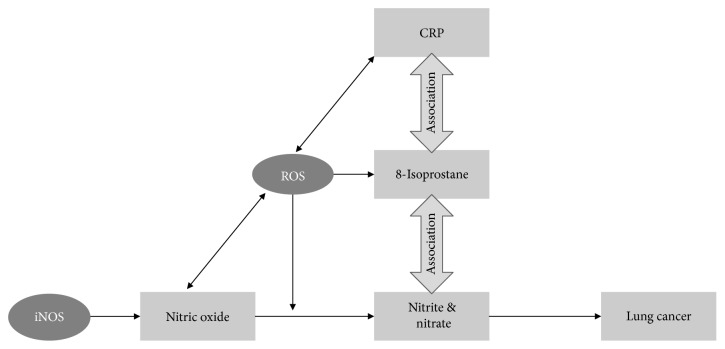
Schematic illustration of observed associations of nitrite/nitrate, 8-isoprostane, and CRP concentrations with lung cancer incidence. Abbreviation: CRP: C-reactive protein; iNOS: inducible nitric oxide synthase; ROS: reactive oxygen species.

Figure 2 illustrates the observed associations of nitrite/nitrate, 8-isoprostane, and CRP levels with each other and with lung cancer incidence and puts them into context with reactive oxygen species (ROS) and NO. Previous studies have shown urinary 8-isoprostane levels and CRP to be associated with lung cancer risk [17, 18]. Nitrite/nitrate levels were associated with 8-isoprostane levels in our study but not with CRP. However, we showed in a previous analysis of the ESTHER study that 8-isoprostane levels and CRP are associated with each other. In the context of these close associations of these oxidative stress and inflammatory biomarkers, the observed independent association of nitrite/nitrate levels with lung cancer incidence was surprising. However, adjustment for other biomarkers of oxidative stress or inflammation (e.g., interleukin-6) might have led to stronger attenuations of the strength of the association and should be investigated in future studies.

The following mechanisms could explain the observed association of nitrite/nitrate concentrations in urine with lung cancer incidence. NO can react with other reactive oxygen species (ROS) which is converted to reactive nitrogen species (RNS), which are subsequently metabolized to nitrite and nitrate [3]. NO can be oxidized to nitrite in the presence of oxygen. Nitrate is produced by the reaction of NO and singlet oxygen, in which peroxynitrite (ONOO^–^) is generated as a midproduct. Peroxynitrite is a strong oxidant and may exert negative effects on DNA, proteins, and lipids initiating cancer cell transition [14, 19]. In addition, RNS/ROS-sensitive pathways may be activated to promote cell growth/proliferation, differentiation, and angiogenesis in cancer [9].

To the best of our knowledge, the associations of NO metabolites and the risk of lung or any other cancer site have not been investigated by prospective, population-based studies before. However, a case-control study nested in a prostate cancer cohort from Sweden observed that high compared to low/negative iNOS immunoreactivity in prostate tumor epithelial cells was associated with a strongly increased 10-year prostate cancer mortality (OR (95% CI), 3.80 (1.45-9.97)) [20]. These findings for prostate cancer suggest that elevated NO production may also be a useful prognostic marker in cancer research, which deserves attention in future studies.

In addition to 8-isoprostane levels, nitrite/nitrate levels were associated with current smoking and a low BMI among cases. This might be explained by the long latency period of lung cancer, and some lung cancer cases diagnosed during follow-up may have already been subclinical at the baseline examination. Lung cancer patients often experience loss of appetite and weight for a long period of time before they are diagnosed [21]. The association of a nitrite/nitrate concentration with current smoking was expected because cigarette smoke itself contains NO, which can cross the alveolar-capillary membrane. NO may also dilate the constricted respiratory tract, making more smoke get into the lung [15]. A previous study demonstrated that smoking led to increased nitrite/nitrate levels in exhaled breath condensate of subjects [21].

Furthermore, we observed that nitrite/nitrate levels were inversely associated with age in the control group, which confirms previous observations in healthy individuals [22]. This inverse association could be explained by the increased plasma concentrations of the NO synthase inhibitor, asymmetric dimethylarginine (ADMA), in older subjects [23]. However, the mechanism for these findings is not clear so far.

It has been shown that NO has pathophysiological effects on asthma and COPD. Elevated NO can lead to nitrosative stress in the airway epithelium, which may be responsible for steroid resistance or ineffectiveness in inflammatory pulmonary diseases [24]. Furthermore, cancer-cell-derived NO may promote cancer-cell invasion, proliferation, and angiogenesis [25]. Therefore, the link between NO and lung cancer suggests that inhibiting NO production might be a potential preventive and/or therapeutic strategy for lung cancer. The major approach to NO inhibition is suppressing NOS activity. A randomized preclinical trial reported that L-NMMA, a competitive NOS inhibitor, decreased lung tumor growth in a mouse model [26]. There is also evidence showing that inhaled steroid treatment reduces exhaled NO as well as the risk of lung cancer [27, 28]. However, the NOS inhibition approaches should be used with caution. Potential side effects, such as endothelial dysfunction, may be caused by inhibiting the NOS [29].

Our study has several strengths. First, a prospective matched case-control study design was used, and subjects with a history of lung cancer before baseline were excluded from the analyses to avoid reverse causality. In addition, cancer cases diagnosed within the first 5 years of follow-up were excluded in a sensitivity analysis to address the lag time of lung cancer development, and the results were consistent with the main results. Second, linkage to cancer registries ensured high certainty regarding lung cancer diagnoses and minimized attrition bias, which often affects cohort studies with long-term follow-up. Third, in order to control for confounding, cases were matched to controls for age, sex, smoking status, and pack-years of smoking, and models were comprehensively adjusted for other potential confounders, including 8-isoprostane levels and CRP. Nevertheless, several limitations in our study need to be considered when interpreting the results. First, the sampling of controls reduces precision and power compared to a cohort design. Second, residual confounding can generally not be excluded in an observational study. Third, the number of cases in each quintile was relatively small and led to a rather low statistical power for quintile comparisons. Fourth, urinary nitrite/nitrate concentrations may also be influenced by a nitrate-rich diet [30, 31]. Although the logistic regression models were adjusted for vegetable consumption and meat consumption frequency, we cannot exclude residual confounding by diet in our analyses because no detailed food frequency questionnaire asking for specific nitrate-rich vegetables was used in our study. Lastly, urine samples were collected only once at baseline. Further studies with repeated measurements are needed because urinary nitrite/nitrate levels might vary during follow-up.

In conclusion, the current study observed that high urinary nitrite/nitrate levels were associated with high lung cancer incidence although the comparison between cases and controls was matched for age, sex, and smoking and controlled for other biomarkers of oxidative stress and inflammation. This suggests an independent mechanism that links pathologically high levels of NO to lung cancer development.

## Figures and Tables

**Table 1:** Baseline characteristics of the incident lung cancer cases and matched controls, the ESTHER study (2000-2014).

Characteristics	Incident lung cancer cases			Controls			*P*_difference_
	*n* (cases)	%	Median (IQR)	*n* (controls)	%	Median (IQR)	
Age (years)	245	—	62 (59-68)	735	—	63 (59-68)	0.885
Sex							
Female	75	30.6		226	30.7	—	
Male	170	69.4		509	69.3	—	
Smoking status							0.937
Never smoker	29	12.1		93	13.0	—	
Former smoker	87	36.2		260	36.2	—	
Current smoker	124	51.7		365	50.8	—	
Pack-years of smoking	221	—	34.8 (19.3-48.0)	624	—	33.8 (14.0-47.4)	0.423
School education (years)							0.077
≤9	198	83.2	—	551	77.0	—	
10-11	19	8.0	—	82	11.4	—	
≥12	21	8.8	—	83	11.6	—	
Physical activity							0.182
Inactive	67	27.6	—	165	22.5	—	
Sedentary	110	45.3	—	351	48.0	—	
Vigorously active	66	27.1	—	216	29.5	—	
BMI (kg/m^2^)							**0.037**
<25	83	33.9	—	202	27.0	—	
25 - <30	103	42.0	—	330	44.2	—	
≥30	59	24.1	—	215	28.8	—	
Meat consumption							0.711
<once/week	81	36.0	—	259	37.4	—	
Once/week	133	54.2	—	369	53.3	—	
>once/week	22	9.8	—	65	9.4	—	
Vegetable consumption							0.338
<once/week	25	10.6	—	110	15.6	—	
Once/week	160	68.1	—	442	62.5	—	
>once/week	50	21.3	—	155	21.9	—	
Asthma							0.292
Yes	21	8.9	—	49	6.8	—	
No	216	91.1	—	671	93.2	—	
Family history of lung cancer							0.709
Yes	27	11.4	—	76	10.5	—	
No	210	88.6	—	646	89.5	—	
CRP (mg/L)	243	—	2.6 (1.3-5.8)	729	—	2.2 (1.1-5.1)	0.164
8-Isoprostane (nmol/mmol creatinine)	240		0.25 (0.18-0.33)	729		0.23 (0.17-0.31)	0.059
Nitrite/nitrate (*μ*mol/mmol creatinine)	245	—	122 (80-206)	733	—	114 (75-170)	0.081

Abbreviation: BMI: body mass index; CRP: C-reactive protein. Note: lung cancer cases and controls were 1 : 3 matched for age, sex, smoking status, and pack-years of smoking.

**Table 2:** Median (IQR) of a nitrite/nitrate concentration according to population characteristics in cases and controls, the ESTHER study (2000-2014).

Characteristics	Cases		Controls	
	*n* (cases)	Nitrite/nitrate (*μ*mol/mmol creatinine)	*n* (controls)	Nitrite/nitrate (*μ*mol/mmol creatinine)
Age (year)				
50-60	79	122.1 (85.2-202.1)	245	122.0 (88.0-182.9)
60-64	62	130.3 (79.4-256.5)	184	120.9 (80.4-187.2)
65-69	60	103.2 (69.7-160.6)	176	98.9 (64.9-158.8)
70-75	44	136.2 (88.7-237.5)	130	97.1 (62.7-147.0)
*Pvalue*		0.323		**<0.001**
Sex				
Female	75	121.5 (67.8-227.0)	226	117.6 (78.8-184.0)
Male	170	120.8 (81.4-181.7)	509	111.2 (71.9-164.3)
*Pvalue*		0.595		0.199
Smoking status				
Never smoker	29	104.7 (60.4-125.5)	93	108.9 (74.7-146.3)
Former smoker	87	112.6 (70.0-172.7)	260	102.2 (64.8-151.4)
Current smoker	124	139.3 (92.8-231.1)	365	127.2 (87.0-188.7)
*Pvalue*		**0.009**		**<0.001**
Pack-years of smoking				
≤15.0	73	107.1 (60.4-167.1)	274	106.9 (71.8-158.1)
15.0 - ≤34.0	59	117.7 (69.4-204.8)	160	114.5 (74.6-162.5)
34.0 - ≤47.5	56	141.7 (89.5-198.6)	148	114.6 (75.9-171.7)
>47.5	57	136.5 (94.5-248.1)	153	122.0 (79.4-197.1)
*Pvalue*		0.071		0.105
Education levels (years)				
≤9	198	122.0 (78.7-195.8)	551	113.7 (74.1-168.6)
10-11	19	112.6 (79.9-235.3)	82	112.7 (67.7-163.5)
≥12	21	145.7 (93.4-250.4)	83	122.7 (92.6-211.9)
*Pvalue*		0.788		0.103
Physical activity				
Inactive	67	121.6 (77.9-193.6)	165	118.6 (76.7-183.5)
Sedentary	110	123.7 (78.7-227.2)	351	110.8 (72.5-170.6)
Vigorously active	66	121.2 (94.1-216.0)	216	118.3 (74.6-167.7)
*Pvalue*		0.846		0.510
BMI (kg/m^2^)				
<25	83	135.8 (85.9-222.0)	199	118.4 (83.2-168.8)
25 - <30	103	134.4 (86.4-229.2)	325	114.3 (72.1-173.8)
≥30	59	98.7 (58.6-170.7)	211	105.0 (70.4-162.5)
*Pvalue*		**0.022**		0.337
Meat intake frequency				
<once/week	81	125.5 (85.0-191.6)	259	114.3 (75.2-177.2)
Once/week	122	112.9 (79.4-207.5)	369	113.8 (74.3-165.7)
>once/week	22	121.8 (64.6-195.8)	65	120.5 (76.2-170.0)
*Pvalue*		0.933		0.877
Vegetable consumption frequency				
<once/week	25	108.5 (71.3-181.7)	108	111.6 (75.7-152.3)
Once/week	160	125.4 (86.1-202.0)	435	113.8 (72.5-163.8)
>once/week	50	105.8 (67.0-188.3)	152	134.6 (80.8-227.3)
*Pvalue*		0.240		0.134
Asthma prevalence				
No	216	121.5 (79.4-200.6)	671	114.0 (74.7-170.0)
Yes	21	107.2 (80.7-229.7)	49	112.7 (78.9-147.0)
*Pvalue*		0.520		0.921
Family history of lung cancer				
No	210	121.2 (79.9-217.7)	646	113.9 (74.7-168.7)
Yes	27	132.3 (71.3-191.6)	76	111.3 (72.0-186.0)
*Pvalue*		0.870		0.907
CRP (mg/L)				
≤1.175	52	114.3 (63.5-236.5)	191	118.0 (78.1-178.3)
1.175 - ≤2.325	59	148.9 (85.9-229.7)	184	108.9 (70.8-162.2)
2.325 - ≤5.140	67	113.0 (89.4-207.5)	177	111.2 (78.2-165.7)
>5.140	65	118.1 (76.0-171.5)	177	114.8 (70.3-174.6)
*Pvalue*		0.520		0.739
8-Isoprostane (nmol/mmol creatinine)				
≤0.175	54	108.3 (69.4-158.0)	189	105.0 (70.5-155.7)
0.174 - ≤0.231	54	112.6 (79.5-193.6)	188	108.4 (73.4-162.5)
0.231 - ≤0.308	59	107.9 (67.1-176.9)	182	114.1 (75.7-175.5)
>0.308	73	167.0 (112.0-267.3)	170	138.9 (88.3-195.9)
*Pvalue*		**0.001**		**0.026**

Abbreviations: BMI: body mass index; CRP: C-reactive protein.

**Table 3:** Associations of nitrite/nitrate concentration quintiles with lung cancer incidence, the ESTHER study (2000-2014).

	Nitrite/nitrate levels (*μ*mol/mmol creatinine)	*n*_case_/*n*_control_	Main model^a^ OR (95% CI)	Sensitivity model 1^b^ OR (95% CI)	Sensitivity model 2^c^ OR (95% CI)
Quintile 1	<66.9	48/147	Ref.	Ref.	Ref.
Quintile 2	66.9 - <97.2	39/147	0.81 (0.59-1.12)	0.81 (0.59-1.12)	0.82 (0.60-1.12)
Quintile 3	97.2 - <134.1	43/147	0.88 (0.65-1.20)	0.89 (0.65-1.21)	0.88 (0.65-1.20)
Quintile 4	134.1 - <192.8	48/147	1.00 (0.74-1.36)	1.01 (0.75-1.37)	1.01 (0.74-1.36)
Quintile 5	≥192.8	67/147	**1.37 (1.04-1.82)**	**1.38 (1.04-1.82)**	**1.36 (1.03-1.80)**

^a^Adjusted for body mass index (BMI), education, family history of lung cancer, asthma, physical activity, and vegetable and meat consumption frequency. In addition, potential confounding by the following factors was controlled by matching age, sex, smoking status, and pack-years of smoking. ^b^Adjusted for variables of the main model+C-reactive protein. In addition, potential confounding by the following factors was controlled by matching age, sex, smoking status, and pack-years of smoking. ^c^Adjusted for variables of the main model+8-isoprostane. In addition, potential confounding by the following factors was controlled by matching age, sex, smoking status, and pack-years of smoking. Note: numbers in bold: statistically significant estimates compared to the quintile 1 (*P* < 0.05).

**Table 4:** Associations of a nitrite/nitrate concentration with lung cancer incidence in a sensitivity analyses excluding lung cancer cases which occurred in the first 5 years of follow-up, the ESTHER study (2000-2014).

	Nitrite/nitrate levels (*μ*mol/mmol creatinine)	*n*_case_/*n*_control_	Main model^a^ OR (95% CI)	Sensitivity model 1^b^ OR (95% CI)	Sensitivity model 2^c^ OR (95% CI)
Quintile 1	<67.7	37/101	Ref.	Ref.	Ref.
Quintile 2	67.7 - <97.4	29/101	0.88 (0.60-1.29)	0.88 (0.602-1.29)	0.88 (0.60-1.29)
Quintile 3	97.4 - <133.7	31/101	0.95 (0.65-1.38)	0.95 (0.66-1.39)	0.95 (0.66-1.39)
Quintile 4	133.7 - <188.8	26/101	0.81 (0.55-1.18)	0.80 (0.55-1.18)	0.81 (0.55-1.19)
Quintile 5	≥188.77	45/100	1.36 (0.97-1.92)	1.36 (0.97-1.92)	1.34 (0.95-1.90)

^a^Adjusted for body mass index (BMI), education, family history of lung cancer, asthma, physical activity, and vegetable and meat consumption frequency. In addition, potential confounding by the following factors was controlled by matching age, sex, smoking status, and pack-years of smoking. ^b^Adjusted for variables of the main model+C-reactive protein (CRP). In addition, potential confounding by the following factors was controlled by matching age, sex, smoking status, and pack-years of smoking. ^c^Adjusted for variables of the main model+8-isoprostane. In addition, potential confounding by the following factors was controlled by matching age, sex, smoking status, and pack-years of smoking.

## Data Availability

The total ESTHER study data cannot be made freely available due to data security regulations. Requests for access to the data used for this publication can be made to the corresponding author, Dr. Ben Schöttker (b.schoettker@dkfz.de).
